# Profiling of patient-specific myocytes identifies altered gene expression in the ophthalmoplegic subphenotype of myasthenia gravis

**DOI:** 10.1186/s13023-019-1003-y

**Published:** 2019-01-29

**Authors:** Melissa Nel, Sharon Prince, Jeannine M. Heckmann

**Affiliations:** 1 Neurology Research Group, Division of Neurology, E8-30, New Groote Schuur Hospital, Department of Medicine, Faculty of Health Sciences, University of Cape Town, Cape Town, 7925 South Africa; 2 Department of Human Biology, Faculty of Health Sciences, University of Cape Town, Cape Town, 7925 South Africa; 3 Division of Neurology, E8-74, New Groote Schuur Hospital, Department of Medicine, Faculty of Health Sciences, University of Cape Town, Cape Town, 7925 South Africa

**Keywords:** Myasthenia gravis, Ophthalmoplegia, Gene expression, Transdifferentiation, Subphenotype, Myotranscriptome

## Abstract

**Background:**

While extraocular muscles are affected early in myasthenia gravis (MG), but respond to treatment, we observe a high incidence of treatment-resistant ophthalmoplegia (OP-MG) among MG subjects with African genetic ancestry. Previously, using whole exome sequencing, we reported potentially functional variants which associated with OP-MG. The aim of this study was to profile the expression of genes harbouring the OP-MG associated variants using patient-derived subphenotype-specific ‘myocyte’ cultures.

**Methods:**

From well-characterised MG patients we developed the ‘myocyte’ culture models by transdifferentiating dermal fibroblasts using an adenovirus expressing MyoD. These myocyte cultures were treated with homologous acetylcholine receptor antibody-positive myasthenic sera to induce muscle transcripts in response to an MG stimulus. Gene expression in myocytes derived from OP-MG (*n* = 10) and control MG subjects (MG without ophthalmoplegia; *n* = 6) was quantified using a custom qPCR array profiling 93 potentially relevant genes which included the putative OP-MG susceptibility genes and other previously reported genes of interest in MG and experimental autoimmune myasthenia gravis (EAMG).

**Results:**

OP-MG myocytes compared to control MG myocytes showed altered expression of four OP-MG susceptibility genes (*PPP6R2*, *CANX*, *FAM136A* and *FAM69A*) as well as several MG and EAMG genes (*p* < 0.05). A correlation matrix of gene pair expression levels revealed that 15% of gene pairs were strongly correlated in OP-MG samples (r > 0.78, *p* < 0.01), but not in control MG samples. OP-MG susceptibility genes and MG-associated genes accounted for the top three significantly correlated gene pairs (r ≥ 0.98, *p* < 1 × 10^− 6^) reflecting crosstalk between OP-MG and myasthenia pathways, which was not evident in control MG cells. The genes with altered expression dynamics between the two subphenotypes included those with a known role in gangliosphingolipid biosynthesis, mitochondrial metabolism and the IGF1-signalling pathway.

**Conclusion:**

Using a surrogate cell culture model our findings suggest that muscle gene expression and co-expression differ between OP-MG and control MG individuals. These findings implicate pathways not previously considered in extraocular muscle involvement in myasthenia gravis and will inform future studies.

**Electronic supplementary material:**

The online version of this article (10.1186/s13023-019-1003-y) contains supplementary material, which is available to authorized users.

## Background

Myasthenia gravis (MG) is a rare antibody-mediated neuromuscular disease in which predominantly acetylcholine receptor (AChR) antibodies target the muscle endplate resulting in fatigable weakness of skeletal muscles. Antibody-mediated complement activation results in muscle endplate damage and ultrastructural changes in all muscle groups, including extraocular muscles (EOMs) [1]. EOMs, which are particularly susceptible to complement mediated damage in MG due to their relative deficiency of complement inhibitors and other factors, are commonly involved early in the disease but typically respond to therapy [2, 3].

Though the incidence of MG in sub-Saharan Africa is comparable to world figures [4], we observe a high frequency of treatment-resistant ophthalmoplegia in this region characterized by severe, persistent eye muscle weakness, which we refer to as OP-MG [5]. In our clinical experience, OP-MG most commonly affects subjects with juvenile onset, but otherwise characteristic AChR antibody-positive MG (i.e. generalized muscle weakness which responds to treatment) [6]. The OP-MG subphenotype results in significant impairment of visual function and ranges from severe paresis of most EOMs to complete paralysis of all EOMs (complete ophthalmoplegia) with ptosis in severe cases. The pathogenesis of the OP-MG subphenotype remains unknown.

We hypothesize that OP-MG might result from excessive complement-mediated damage of muscle endplates coupled with impaired regeneration in the EOMs [5]. Previously we found that a subset of OP-MG individuals harbour functional regulatory region variants in the decay accelerating factor (*DAF* or *CD55*) [7] and transforming growth factor beta 1 (*TGFB1*) genes [8] which lower their respective expression levels. Impaired upregulation of DAF*,* a complement regulatory protein which mitigates complement activation, and TGFB1, a prominent myokine which also upregulates DAF expression in the orbital environment [9], suggests that potentiated complement mediated injury and altered healing of the EOMs may contribute to OP-MG pathogenesis.

We also performed extended whole exome sequencing (WES) in a well characterized cohort of OP-MG and control MG individuals, all AChR antibody-positive and differing only by the responsiveness of their EOMs to standard therapy. This approach identified a number of potentially functional OP-MG associated regulatory region variants which were more common in OP-MG compared to control MG individuals [10]. The gene list containing these candidate variants was filtered and putative OP-MG susceptibility genes were prioritised based on whether their expression was detected in a RNA microarray of normal human extraocular muscle tissue [11].

Because of the difficulty in obtaining relevant EOM tissue, we developed a phenotype and MG disease specific muscle cell culture model through transdifferentiation of primary dermal fibroblasts into myocytes. The focus of the present study was to compare the expression of relevant genes in OP-MG vs control MG samples using this model of the myotranscriptome. Relevant genes included those harbouring OP-MG susceptibility variants and additional genes differentially expressed in MG or experimental autoimmune MG (EAMG) based on published studies.

## Material and methods

### OP-MG and control MG definition

Sixteen individuals (10 OP-MG and 6 control MG) all with African-genetic ancestry (black or mixed-African ancestry as previously described [5, 10]) and generalized AChR-antibody positive MG with prolonged follow-up at the myasthenia gravis clinic at Groote Schuur Hospital, University of Cape Town, South Africa donated skin biopsies. OP-MG was defined as individuals with otherwise characteristic generalized MG, but in whom the EOMs remained treatment-resistant whereas control MG individuals may have had typical EOM weakness as part of their initial MG presentation, but responded to therapy and have since remained free of persistent extraocular muscle weakness [5, 8]. There was no significant difference (*p* > 0.05) in black and mixed-African ancestry proportions, age at MG onset, years of follow up or age at skin biopsy between the OP-MG and control MG groups. There was a higher proportion of female subjects in the control MG compared to the OP-MG group (100% vs 40%, *p* = 0.033) (Table 1).

**Table 1:** Clinical characteristics of the 16 skin biopsy donors by subphenotype

	control MG	OP-MG	*p* value
female n (%)	6 (1)	4 (0.40)	0.033
male n (%)	0	6 (0.60)	
M/A n (%)	4 (0.67)	5 (0.50)	0.633
B n (%)	2 (0.33)	5 (0.50)	
age at MG onset (yrs), mean (IQR)	21 (18–24)	18 (11–22)	0.576
years of follow up, mean (IQR)	13 (5–10)	15 (10–17)	0.679
age at skin biospy (yrs), mean (IQR)	31 (25–30)	30 (23–34)	0.947

*M/A* mixed-African ancestry, *B* black/indigenous African ancestry, *yrs*. years, *IQR* interquartile range. Continous data was compared with an unpaired Student’s t-test while categorical data was compared with Fisher’s exact test

### Ethics and consent

The study was approved by the University of Cape Town Health Sciences Faculty Research Ethics committee (HREC 257/2012) and all individuals (or their parents if < 18 years) signed informed consent to participate.

### Skin biopsies and primary dermal fibroblast culture

Skin punch biopsies (3 mm full thickness) were obtained from the scapular area of each donor. The epidermis and superficial dermal layer was separated from the subcutaneous tissue, manually minced with surgical blades and cultured under sterile coverslips (explant method) in 35 mm dishes with growth medium (high glucose Dulbecco’s modified Eagle’s medium (DMEM) + 10% foetal bovine serum + 1% penicillin/streptomycin (P/S)) until fibroblasts emerged.

### Development of subphenotype-specific myocyte models

To compare gene expression between OP-MG and control-MG subphenotypes, we developed a muscle cell culture model for each subject. Briefly, 2 × 10^5^ dermal fibroblasts (passage 4) were seeded in 6 cm dishes coated with 0.1 mg/ml Matrigel® in 4 ml growth medium and incubated overnight at 37 °C and 5% CO_2_. The next day the fibroblasts, at 80–90% confluency, were transduced with a RGD fiber modified adenovirus containing a human MyoD transgene and expressing a green fluorescent protein (GFP) reporter (Ad(RGD)-MyoD-GFP) (VectorBiolabs, Philadelphia, USA) at a multiplicity of infection (MOI) of 200. This achieved > 90% transduction efficiency (%GFP+ fibroblasts determined by FACS analysis) (data not shown). Transduced fibroblasts were maintained in differentiation medium (DMEM + 5% horse serum + 1% P/S) and differentiated for either 48 h (early muscle model) or 5 days (late muscle model) to generate myocytes.

After 5 days of differentiation, myocytes showed morphological features of myogenic differentiation including widespread immunostaining of cells with an MF-20 antibody that recognizes all isoforms of sarcomeric myosin (data not shown). However, contrary to the skeletal muscle differentiation program in vivo, not all trans-differentiated dermal fibroblasts exhibited branched, multinucleated myotube formation. The term ‘myocyte’ rather than ‘myotube’ was therefore used to refer to our muscle cell culture model since the morphological features of complete terminal differentiation were not observed.

**Fig. 1:**
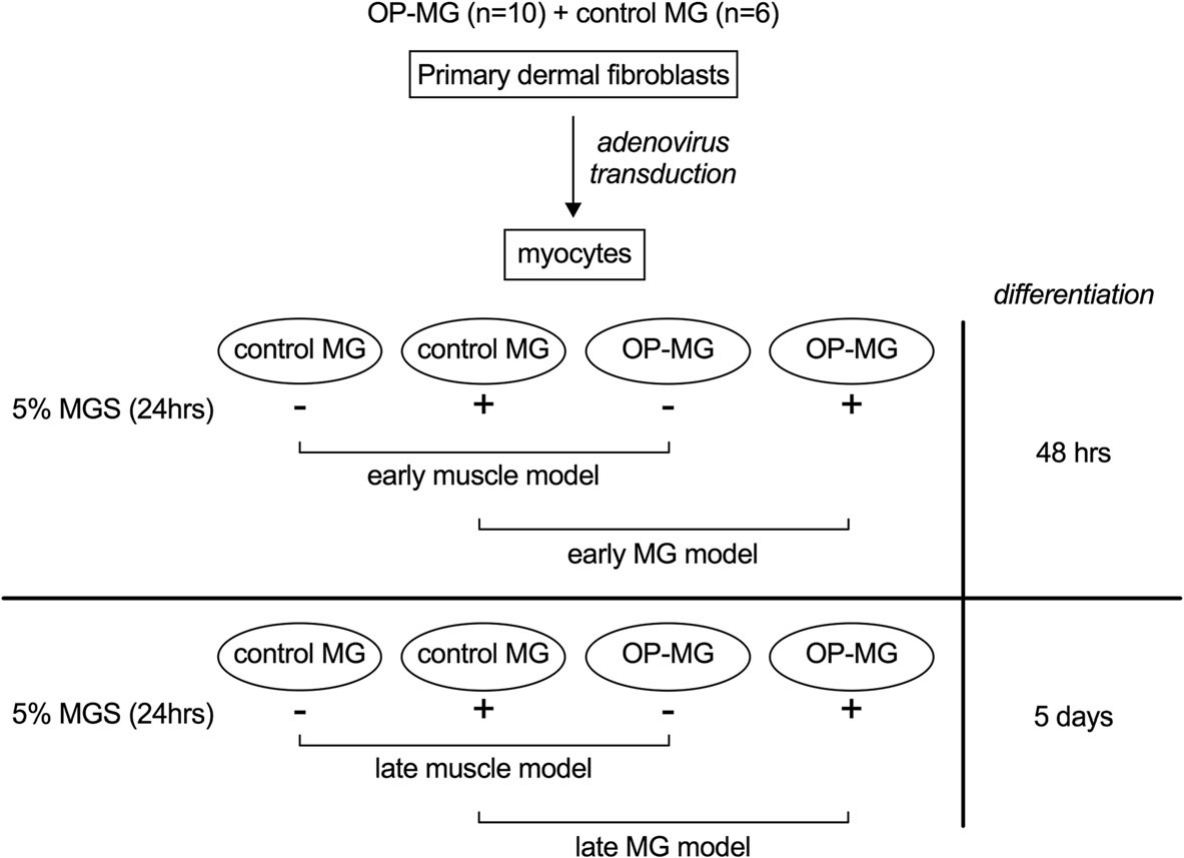
Experimental design. Primary dermal fibroblasts from OP-MG (*n* = 10) and control MG (*n* = 6) donors were transduced with MyoD-adenovirus and differentiated into myocytes for either 48 h (early muscle model) or 5 days (late muscle model). At each differentiation time point, myocytes from each subphenotype were either left untreated or stimulated with 5% MG sera for 24 h (MG model) before RNA was harvested for analysis of gene expression by quantitative PCR

To mimic patient-specific MG-induced muscle pathway responses in vitro*,* we stimulated 48 h and 5 days differentiated myocyte cultures with 5% homologous treatment-naïve AChR-antibody positive MG sera for 24 h before harvesting RNA (early and late MG model) (Fig. 1). The sera sample was sourced from an AChR antibody-positive, treatment-naive MG patient with generalized myasthenia and severe extraocular muscle involvement.

### RNA extraction, quantification and quality control

RNA was extracted from myocytes (*n* = 64) using the HighPure RNA extraction kit (Roche) according to the kit protocol. RNA concentration and purity were determined using the Nanodrop® ND1000 spectrophotometer (Thermo Scientific). All RNA samples had concentrations > 40 ng/μl and ratios within the recommended ranges (A260/280 = 1.8–2.0; A260/230 > 1.7). RNA sample integrity was determined using the Agilent Bioanalyzer Eukaryote Total RNA Nano assay (Agilent). 57/64 samples had a RNA integrity number (RIN) > 7 while the remaining 7/64 samples had a RIN ≥5 which is still acceptable for downstream qPCR analysis [12].

### Custom gene expression array

**Fig. 2:**
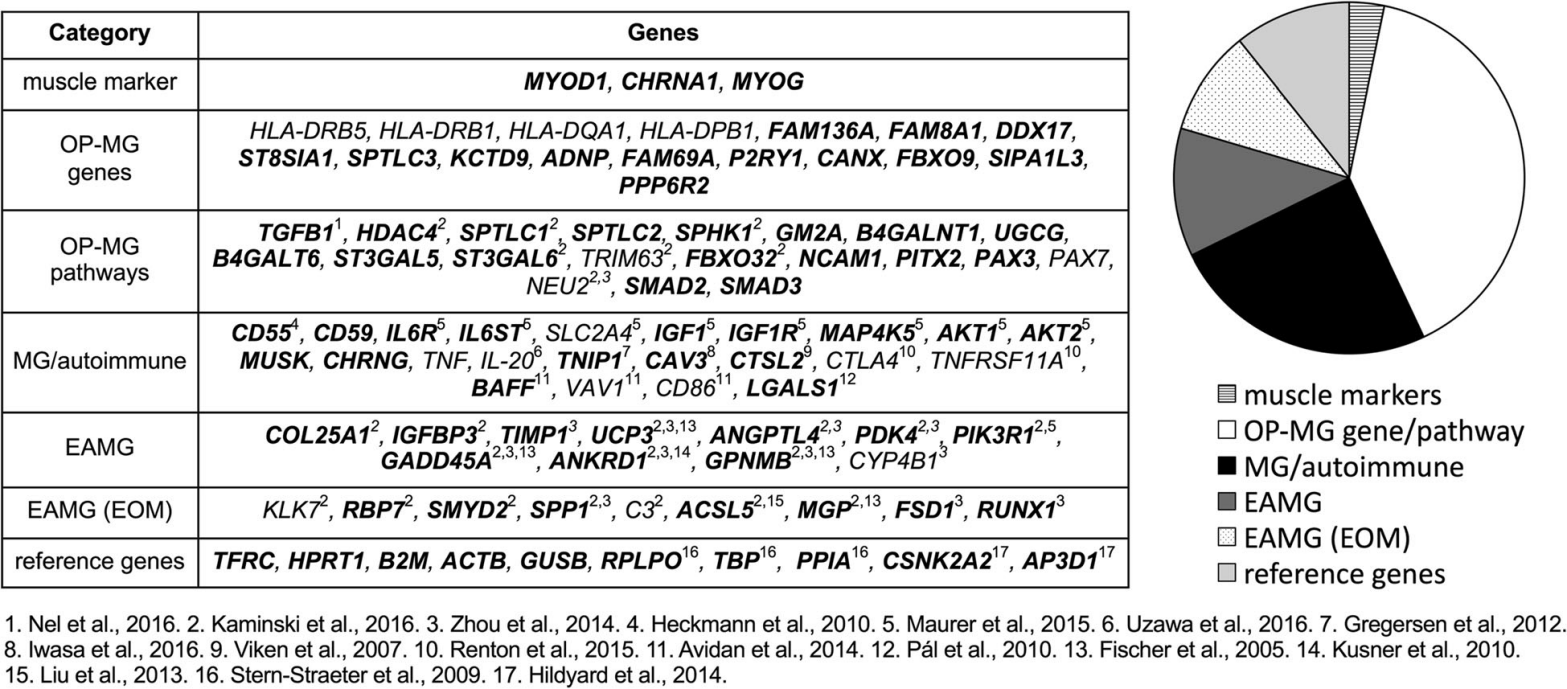
Genes profiled in the expression array grouped according to categories. Expressed genes (Cq < 35 in all samples) are indicated in bold. “OP-MG genes” refers to genes containing OP-MG susceptibility variants and “OP-MG pathways” refers to genes in OP-MG susceptibility pathways [10], MG = myasthenia gravis, EAMG = experimental autoimmune myasthenia gravis, EOM = extraocular muscle

Gene expression profiling of 93 genes and 3 RNA quality controls using proprietary assays (primer sequences not available) was performed using custom 384 well RT^2^ Profiler PCR array plates (Qiagen) at the Centre for Proteomic and Genomic Research (CPGR), Cape Town, South Africa. Figure 2 shows the 93 genes profiled in the expression array grouped according to various categories which is largely based on their association with the OP-MG subphenotype and/or their involvement in biological processes with potential relevance to OP-MG pathogenesis. “Muscle markers” (*n* = 3) includes genes which are specific to the myotranscriptome. “OP-MG genes” (*n* = 17) includes susceptibility genes containing variants suggestive of association with OP-MG (*p* < 0.055) previously identified by WES [10]. “OP-MG pathways” (*n* = 20) includes genes which are functionally related to the OP-MG genes identified by WES. These pathway candidates were selected largely from panels of differentially expressed genes identified through muscle expression profiling studies in passive and active transfer animal models of MG [13, 14], with a particular focus on genes with differential expression patterns in EOM (as opposed to limb muscle). “MG/autoimmune” (*n* = 23) includes genes harboring MG associated variants identified through candidate gene and genome wide association studies and genes which were differentially expressed in muscle tissue from MG patients compared to healthy controls. “EAMG” (*n* = 11) includes a selection of genes which were shown to be differentially expressed in experimental autoimmune MG (EAMG) across all muscle groups while “EAMG EOM” (*n* = 9) includes a selection of genes from EAMG studies which were differentially expressed in EOM as opposed to limb muscle. “Reference genes” (*n* = 10) includes a gene panel included for normalization of target gene expression levels.

### Quantitative real-time PCR

400 ng total RNA was reverse transcribed to cDNA using the RT^2^ First Strand Kit (Qiagen) according to the manufacturer’s specifications. Quantitative PCR was performed on the cDNA samples using RT^2^ SYBR Green Mastermix (Qiagen) on the 7900HT Fast Real-Time PCR System (Applied Biosystems). A genomic DNA control (GDC), reverse transcription control (RTC) and positive PCR control (PPC) were included for each sample. All the C_q_ values for these controls were within the acceptable reference ranges.

### Selection of reference genes

Given the heterogeneity of the RNA samples (OP-MG vs control MG subphenotype, potential variability in the degree of myogenic differentiation, untreated vs MG sera exposure) and in accordance with the Minimum Information for Publication of Quantitative Real-Time PCR Experiments (MIQE) guidelines [15], we screened a panel of 10 reference genes for their expression stability in all 64 RNA samples. These included 5 reference genes commonly used in the literature in a wide variety of tissue contexts (*TFRC, HPRT1, B2M, ACTB, GUSB)* and 5 reference genes which have validated expression stability during normal and diseased cell culture models of myogenesis (*RPLP0, TBP, PPIA, CSNK2A2, AP3D1)* [16, 17]*.* Three methods were used to comprehensively assess the stability of each reference gene: 2^-Cq^ method [18], geNorm [19] and BestKeeper [20]. While the expression of all 10 candidate reference genes was similar across all 64 samples (Cq SD < 1), subgroup analysis enabled the identification of ideal candidates which is necessary to detect small differences in target gene expression. The early and late muscle models were used to assess the impact of differentiation on reference gene stability. The impact of MG sera treatment on reference gene stability was performed separately for the early (48 h) and late (5 days) models. The results of this analysis are summarized in Additional file 1: Table S1 and Figure S1. For subphenotype comparisons in the muscle and MG models, target gene expression levels were normalized to *RPLP0* and *B2M* for the early model and *AP3D1* and *CSNK2A2* for the late model. For comparisons between the early and late muscle models, target gene expression levels were normalized to *GUSB* and *TFRC*.

### Data analysis

#### Differential CHRNA1 isoform expression in myocytes by subphenotype

*CHRNA1* encodes the alpha subunit of the acetylcholine receptor and is transcribed as two major muscle isoforms (P3A+ and P3A-), which are distinguished by the inclusion or exclusion of an additional exon P3A. To determine the expression ratio of these two *CHRNA1* transcripts, standard curves were generated for two *CHRNA1* primer pairs which amplified total *CHRNA1* (P3A+ and P3A-) or only the P3A+ isoform. These were used to interpolate the absolute *CHRNA1* transcript numbers and the ratio P3A+:(P3A+ and P3A-) was used to calculate the % P3A+ isoform expression in myocytes according to the method described by Masuda et al. [21].

#### Differential gene expression analysis

Raw C_q_ values were analysed in Microsoft® Excel for Mac. Genes with an undetermined Cq value in ≥1 sample were excluded from the analysis. Differential gene expression between control MG and OP-MG was assessed independently for the four sepafrate experimental models (early muscle model, early MG model, late muscle model, late MG model) according to the method described by Schmittgen and Livak [18]. Individual data points were calculated as 2^-∆Cq^, where ∆Cq = target gene Cq – reference gene Cq. For each subphenotype group (control MG and OP-MG), the mean and SD of these data points was used to calculate a fold change in gene expression (mean OP-MG 2^-∆Cq^/ mean control MG 2^-∆Cq^). The 95% confidence interval (CI) of the fold change was calculated using the Graphpad online calculator (https://www.graphpad.com/quickcalcs), which is based on Fieller’s theorem [22]. To examine the effect of MG sera on gene expression, 2^-∆Cq^ values for each group (untreated and MG sera treated) were compared according to the same method for both the early and late models. For normally distributed data the Student’s t-test was used to assess whether gene expression differences were statistically significant; unpaired two-tailed test for OP-MG vs control MG comparisons and paired two-tailed test for MG sera treated vs untreated comparisons (since the treated and untreated sample were paired for each individual). If data was not normally distributed (Shapiro-Wilk normality test *p* < 0.05), the Mann-Whitney test was used for comparisons. Uncorrected *p* values are presented with significance set at *p* < 0.05.

#### Differential gene correlation analysis

As a secondary analysis, and after excluding genes with an undetermined Cq value in ≥1 sample, the correlation in ∆C_q_ values for every possible target gene pair was determined for each subphenotype group (control MG and OP-MG) in each of the four separate experimental models (early muscle model, early MG model, late muscle model, late MG model) using RStudio version 1.0.136. The linear correlation between gene pairs was calculated by computing a Pearson correlation co-efficient (r) using the rcorr function in the Harrel Miscellaneous (Hmisc) R package. The statistical significance of the linear correlation of gene pairs is approximated by *p* values using the t or F distributions. *P*-values were adjusted using the Benjamini-Hochberg procedure (FDR < 0.01). To aid visualization of differential gene correlation by subphenotype, correlation matrices were constructed using the corrplot function in R.

## Results

### Gene expression in myocytes

Expressed genes were defined as those with Cq < 35 in all samples (Fig. 2, indicated in bold) (see Additional file 1: Table S2). The following genes were expressed in both early and late model myocytes: 3/3 muscle markers, 13/17 OP-MG genes (all selected genes excluding HLA genes), 17/20 genes in OP-MG pathways, 16/23 ‘MG/autoimmune genes’, 10/11 EAMG genes, 7/9 EAMG (EOM) genes and 10/10 reference genes.

### Myocytes express muscle specific genes

To validate the myotranscriptome and ensure that any detectable differences in target gene expression levels between control MG and OP-MG myocytes reflect the subphenotypic myotranscriptome signatures and not underlying differences in the degree of myogenic differentiation, we sought to determine the levels of 3 ‘muscle markers’ (*CHRNA1, MYOD1, MYOG)* at both early (48 h) and late (5 days) differentiation time points. *MYOD1* and *MYOG* encode muscle-specific transcription factors.

**Fig. 3:**
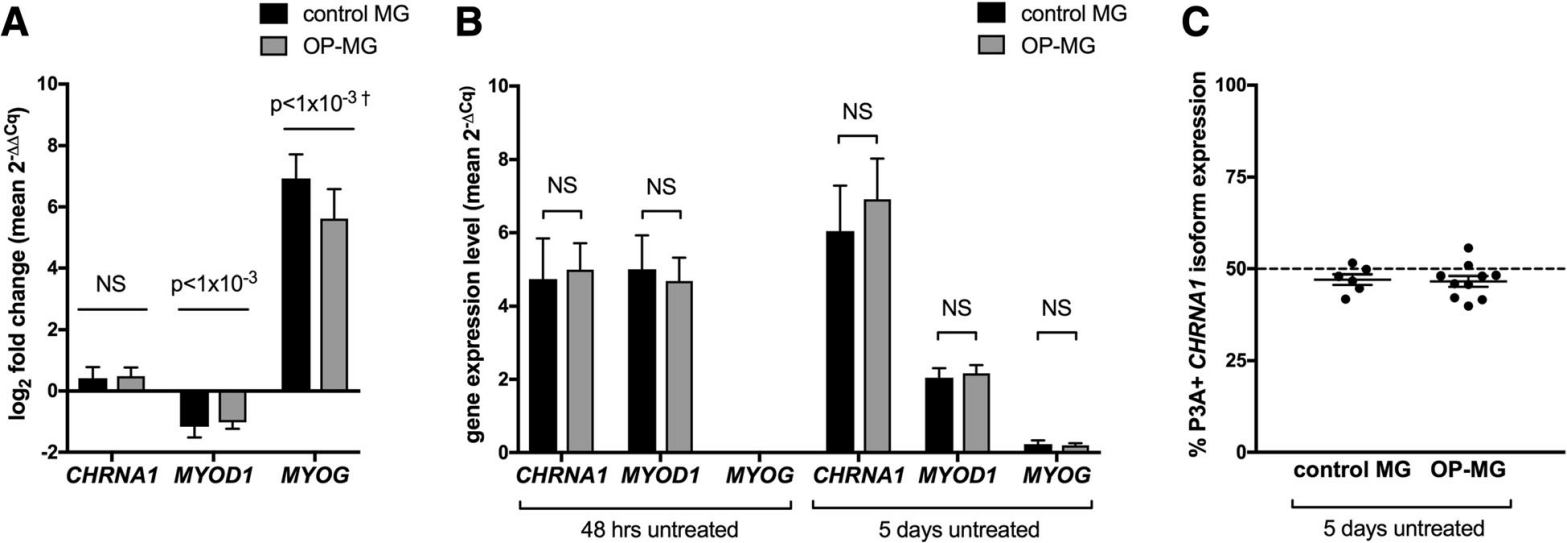
**a** and **b**. Expression of muscle gene transcripts in myocytes by subphenotype in early (48 h) and late (5 days) differentiation models. RNA was extracted from untreated control MG (*n* = 6) and OP-MG (*n* = 10) myocytes after 48 h and 5 days of differentiation as described. For each differentiation time point, expression levels of *CHRNA1*, *MYOD1* and *MYOG* target genes were determined using relative quantification (2^-∆Cq^) where ∆C_q_ represents target gene Cq – average *GUSB/TFRC* Cq (the reference genes which were not influenced by prolonged differentiation of myocytes). **a** Combined log_2_ fold change for both subphenotypes (mean 2^-∆∆Cq^, where ∆∆Cq represents 5 days ∆Cq - 48 h ∆Cq) were compared to assess differences in gene expression levels between the early and late differentiation models. **b** Comparison of gene expression levels (2^-∆Cq^) between subphenotypes in the early and late differentiation models. **c** *CHRNA1* P3A+ isoform expression in OP-MG and control MG myocytes represents in vivo muscle splicing signatures. RNA was extracted from control MG (*n* = 6) and OP-MG (*n* = 10) myocytes after 5 days of differentiation as described. qPCR was performed using two sets of primers for *CHRNA1*: 1 set which recognizes total *CHRNA1* transcripts (P3A+ and P3A-) and another which is specific for P3A+ transcripts. Cq values were used to interpolate absolute transcript numbers from standard curves, then the ratio of P3A+:(P3A+ and P3A-) was calculated for each sample (expressed as %). Error bars show mean and SEM. Student’s t test was used for comparisons where the data was normally distributed, otherwise Mann-Whitney test was used (†) where Shapiro-Wilk normality test *p* < 0.05

In keeping with the transcriptional events which orchestrate myogenesis in vivo, myocytes express muscle specific genes which are undetectable in dermal fibroblasts (data not shown) and show dynamic changes in expression as differentiation progresses from 48 h to 5 days: *MYOD1* ≈ 2-fold downregulated (*p* < 1 × 10^− 3^), *MYOG* ≈160-fold upregulated (*p* < 1 × 10^− 3^) (Fig. 3a). Importantly, there were no differences in the expression of these three muscle specific genes between control MG and OP-MG in both the early and late models indicating a similar degree of myogenic differentiation in both subphenotypes) (Fig. 3b).

In addition, the *CHRNA1* P3A+:P3A- transcript ratio in both control MG and OP-MG myocytes was similar in both subphenotypes (≈50%) (Fig. 3c) and to in vivo muscle splicing patterns in normal [23] and MG samples [24].

### MG sera induces gene expression changes in the myocyte model which are consistent with those in EAMG

**Fig. 4:**
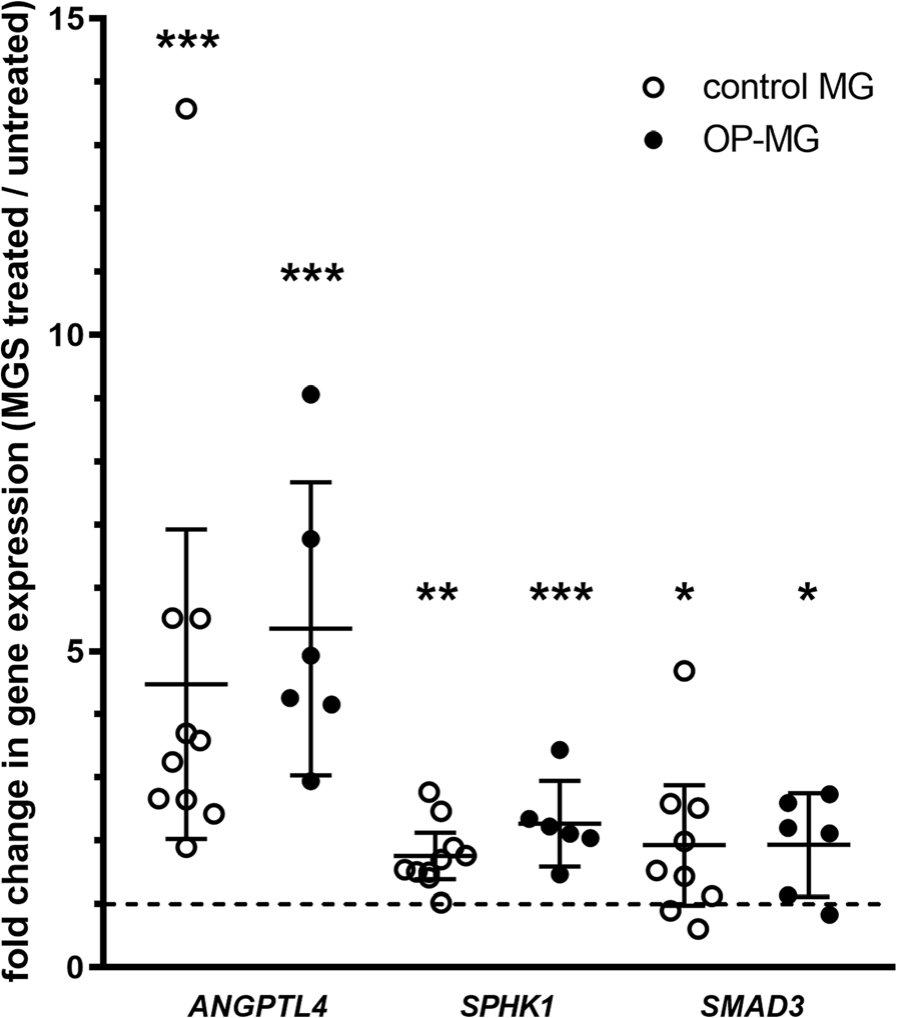
MG sera induces gene expression changes in patient-derived myocytes. RNA was extracted from untreated and MG sera (MGS) treated control MG (*n* = 6) and OP-MG (*n* = 10) myocytes after 48 h differentiation as described. Target gene expression levels were determined using the custom qPCR gene expression array and a fold change in gene expression was calculated (MG sera treated/untreated) for each gene following normalization. Genes with statistically significant (*p* < 0.05) fold changes (> 1.5 up or downregulated) for both control MG and OP-MG are shown. Error bars show mean and 95% CI. Student’s paired t-test was used to compare gene expression levels (MGS vs untreated) for each subphenotype. *p < 0.05, ** *p* < 0.01, *** *p* < 1 × 10^− 3^. 1 datapoint has been excluded from the graph as it lies beyond the y-axis limits

To induce MG-specific pathway responses we stimulated myocytes with 5% MG sera. The top upregulated transcripts (> 1.5-fold) in response to MG sera were similar in control MG and OP-MG myocytes in the early model representing 48 h differentiated myotubes treated with MG sera (*ANGPTL4* ≈ 4-fold upregulated *p* < 1 × 10^− 3^, *SPHK1* ≈ 2-fold upregulated *p* < 0.01, *SMAD3* ≈ 2-fold upregulated *p* < 0.05) (Fig. 4). In previous EAMG studies, *ANGPTL4* was the highest upregulated transcript across 3 muscle groups (limb, diaphragm and EOM) and also expressed at the highest level in EOM [13, 14]. Although *SPHK1* was included in the array as an OP-MG pathway gene, it was also found to be upregulated in EOM in EAMG models [13, 14]. Taken together, this suggests that our ‘MG model’ captures some of the gene expression signatures associated with EAMG and supports its use as a model to profile OP-MG pathways. In contrast, the 5-day differentiated model did not show any significant gene expression changes in response to MG sera.

### Control MG and OP-MG myocytes show different gene expression profiles at basal levels and following exposure to MG sera

**Fig. 5:**
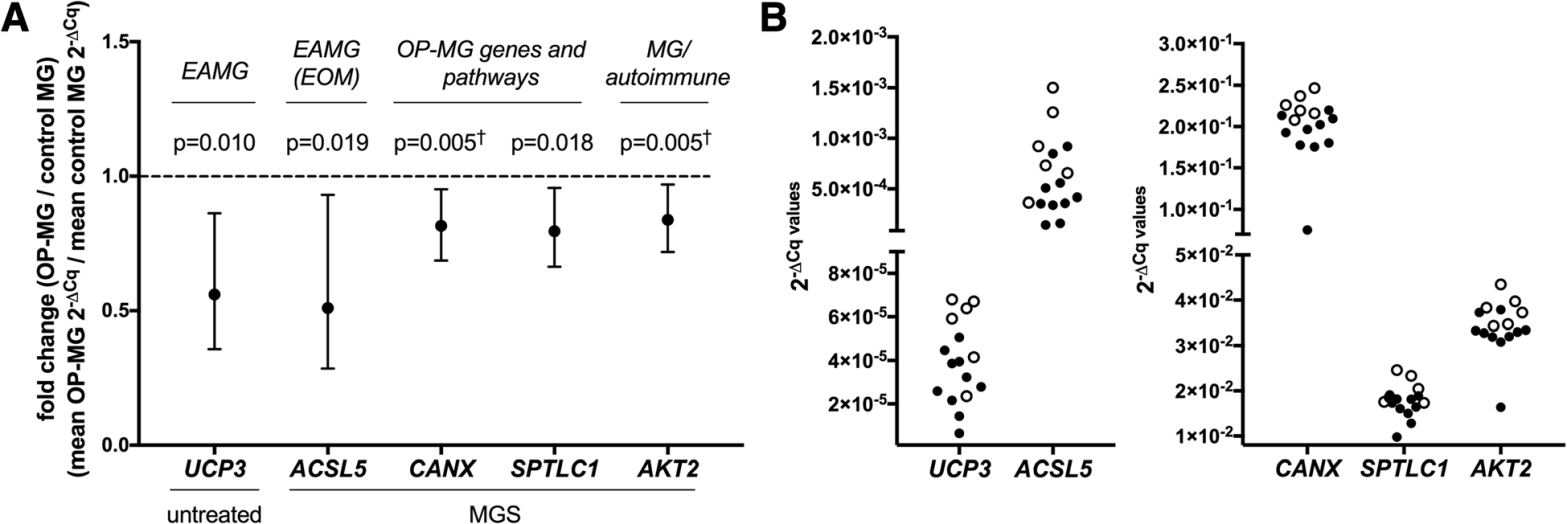
Control MG and OP-MG myocytes show different gene expression profiles. RNA was extracted from untreated and MG sera (MGS) treated control MG (*n* = 6) and OP-MG (*n* = 10) myocytes after 48 h differentiation as described. Target gene expression levels were determined using the custom qPCR gene expression array and a fold change in gene expression (OP-MG/control MG) was calculated for each gene in the early untreated and MGS treated models. Genes with statistically significant (*p* < 0.02) fold changes are shown and the remaining genes (0.02 < *p* < 0.05) are shown in Additional file 1: Figure S2. **a**. shows the fold change as an average of OP-MG/control MG samples (error bars show mean and 95% CI) and **b**. shows the 2^-∆Cq^ values for each sample (open circles = control MG, closed circles = OP-MG). Student’s t test was used for comparisons where the data was normally distributed, otherwise Mann-Whitney test was used (†) where Shapiro-Wilk normality test *p* < 0.05

We found the expression of 14 genes (from all 5 gene categories) differed between OP-MG and control MG myocytes (> 1.5-fold, *p* ≤ 0.041, Fig. 5 and Additional file 1: Figure S2). Seven of the 14 differentially regulated genes were either OP-MG genes (*n* = 4: *PPP6R2*, *CANX*, *FAM136A* and *FAM69A*) or genes in OP-MG pathways (*n* = 3: *PAX3*, *SPTLC1*, *UGCG*). Most differences in gene transcript levels between the two subphenotypes were detected in the early muscle model in response to MG sera where *ACSL5, CANX*, *SPTLC1* and *AKT2* genes had lower expression in OP-MG myocytes compared to controls (*p* < 0.020) (Fig. 5).

### The early MG model showed different gene expression correlations by subphenotype

In addition to identifying differences in gene expression levels between the cell models derived from the two subphenotypes, we also investigated whether we could distinguish the two subphenotypes based on a correlation analysis of gene pair expression levels. This is relevant as differential gene co-expression, particularly in the absence of detecting differentially expressed genes, can be an informative signal to differentiate diseased from non-diseased samples [25] which may identify novel disease-related genes and pathways [26].

**Fig. 6:**
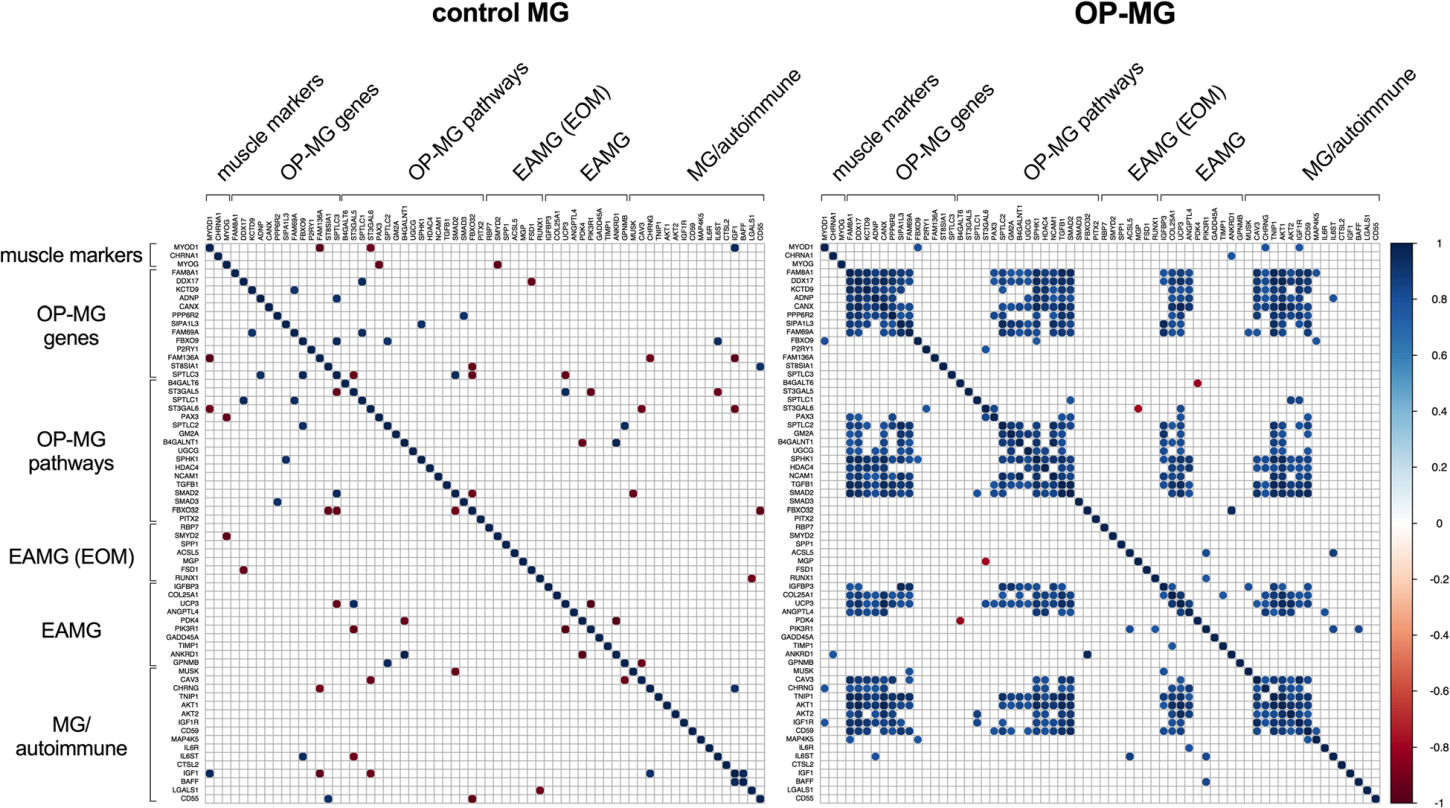
Correlation between gene pair expression levels differs in control MG and OP-MG myocytes. For expressed genes (*n* = 66), the correlation in ∆C_q_ values for every possible target gene pair was determined for each subphenotype group (control MG and OP-MG) in the early MG model using the Pearson correlation co-efficient (r). Statistically significant correlations (unadjusted *p* < 0.01) are shown as a matrix. The color key indicates the strength of positive (blue gradient) and negative (red gradient) correlations. Genes are grouped according to their selection category for the custom qPCR array

In the early MG model, we found that a subset of gene pairs (*n* = 328, 15%) were highly positively correlated among OP-MG samples (r > 0.77, unadjusted *p* < 0.01), both within and between gene categories (Fig. 6). These positive intra- and inter-correlations of gene pair expression levels were evident as co-expression modules within a correlation matrix of gene pair expression levels. In contrast, control MG samples showed few, isolated, mostly negative gene pair correlations.

We applied the Benjamini-Hochberg procedure to identify the most highly correlated gene pairs among OP-MG samples (*n* = 100, r > 0.90, FDR < 0.01). Cross-correlations between OP-MG genes, genes in OP-MG pathways and MG/autoimmune genes accounted for 59 of the gene pairs in this group (37 OP-MG gene/pathway ~ MG/autoimmune gene pairs and 22 OP-MG gene ~ OP-MG pathway gene pairs). While the high number of correlated OP-MG gene and OP-MG pathway genes is expected, the fact that a larger number of OP-MG and MG/autoimmune genes are correlated suggests that significant crosstalk exists between OP-MG and MG pathways. For example, correlations between OP-MG susceptibility genes (*CANX*, *DDX17*, *TGFB1*) and MG genes (*TNIP1*, *AKT1*) accounted for the top three significantly correlated gene pairs (r ≥ 0.98, *p* < 1 × 10^− 6^).

## Discussion

Because of the difficulties in obtaining EOM tissue, we developed an in vitro muscle model, at two differentiation time points, to compare MG subphenotype-specific ‘myo’-transcriptomic responses to active MG sera by interrogating the expression of previously reported OP-MG genes and genes in related pathways. The early differentiation model (48 h) exhibited “myoblast type” gene expression patterns (high levels of *MYOD1*) while the late differentiation model (5 days) model exhibited “myocyte type” gene expression patterns (low levels of *MYOD1* and high levels of *MYOG* which induces the expression of terminal differentiation genes) [27]. Using these models we found evidence of different muscle transcript expression dynamics between the OP-MG and control MG derived myocytes which could represent functional differences in gene expression networks.

We detected expression differences in four OP-MG genes (identified by WES) between OP-MG and control MG myocytes (Fig. 5; Additional file 1: Figure S2): *PPP6R2* and *CANX* in the early model and *FAM136A* and *FAM69A* in the late model. These genes harbour putative 3’UTR OP-MG susceptibility variants which may alter microRNA binding in OP-MG subjects to either increase or decrease their expression levels. Notably, OP-MG genes were prioritised based on their expression in EOM since the OP-MG subphenotype specifically involves EOM rather than limb muscle. It may be reasonable to conclude that our model may not have been adequate to capture differences in the expression of other OP-MG genes if this is only altered in the unique EOM transcriptome. Similarly, of the EAMG (EOM) genes included in the array due to their differential expression in EOMs in experimental MG models [13, 14], only one (*ACSL5*) showed significant changes in gene expression between the OP-MG and control MG myocytes in response to MG sera. Overall, the most informative model was the early muscle model (48 h differentiation) in response to MG sera, which showed more gene expression differences between the two subphenotypes than the late model. This may suggest that early myogenesis regenerative events are impaired in OP-MG individuals following MG induced muscle damage.

Human EOMs, compared to other skeletal muscles, have significantly more mitochondria due to their energy requirements. In the early model the expression of *UCP3*, which encodes a mitochondrial uncoupling protein, was significantly downregulated in OP-MG compared to control MG myocytes. UCP3 may reduce the production of reactive oxygen species (ROS) and protect mitochondria under conditions of EAMG [13] which suggests that lower basal levels of UCP3 in OP-MG myocytes may impair this protective mechanism.

We previously interrogated CD55 (DAF) due to its critical role in muscle endplate damage in myasthenia [28] and its relatively lower expression in the EOMs compared to limb muscle [3, 28]. Here we found an upregulation of *CD55* expression in OP-MG myocytes compared to control MG which was similar to our previous observations in lymphoblastoid cell lines from the two subphenotypes; however, previously we showed that CD55 was significantly repressed in response to lipopolysaccharide (representing an immune stimulus) in OP-MG derived cells [7].

Despite the limitation of not having EOMs to interrogate, we found in OP-MG myocytes, but not control MG, different patterns of gene co-expression (inferred from expression correlation) between the unbiased OP-MG genes/ OP-MG pathways and genes known to be involved in MG/autoimmune and EAMG pathways. For example, 53% (16 of 30) of the expressed OP-MG genes showed significant cross-correlations of expression levels (FDR < 0.01) with 42% (11 of 26) of the EAMG and MG/autoimmune genes in OP-MG myocytes (Fig. 6). This observation of gene expression correlation across a group of individuals (such as OP-MG cases) may suggest that these genes are functionally related [24], perhaps within the same pathway(s).

For presentation of the gene co-expression data, we grouped genes in the matrix (Fig. 6) by biological function or pathway. For example, *ST8SIA1 and SPTLC3* (OP-MG genes identified in our previous WES study [10]) encode enzymes involved in gangliosphingolipid biosynthesis and as such they were grouped with other candidates in this pathway. This visual organisation highlighted the fact that genes in the same pathway were strongly correlated in OP-MG, but not control MG. Although gangliosphingolipids are not known to play a role in MG they are critical in maintaining the integrity of the muscle endplate through their formation of lipid rafts which stabilize membrane bound receptors and signaling molecules such as AChR [29], GP130 (or IL6ST) [30], CD55 (DAF) and CD59 [31, 32]. The initial reaction in sphingolipid synthesis requires the enzyme serine palmitoyltransferase (SPT) which is encoded by *SPTLC1*, *SPTLC2*, and *SPTLC3* genes. Interestingly, *SPTLC1* expression was lower in OP-MG compared to control MG myocytes which may suggest that the sphingolipid synthesis pathway is impaired in OP-MG myocytes in response to MG sera.

Several genes related to IGF1-signalling were included in the array as this pathway has already been implicated in MG [33], though not specifically considered in the pathogenesis of EOM involvement in MG. Interestingly, the expression of OP-MG genes strongly correlated with several genes from this pathway (*IGF1*, *AKT1*, *AKT2*).

Since we used a transdifferentiation model, the snapshot of the myotranscriptome obtained in both the muscle- and MG-models may not accurately capture the biological signal or the magnitude of putative signals of altered gene/pathway function in OP-MG EOMs, even if the effect sizes are substantial. Nevertheless, the MG-muscle model showed expression differences in several functionally related genes between OP-MG and controls which provides a basis for exploring these putative pathogenic pathways in future work.

## Conclusion

Using a surrogate cell culture model our findings suggest that muscle gene expression and co-expression differ between OP-MG and control MG individuals in response to MG sera. These findings implicate pathways not previously considered in extraocular muscle involvement in myasthenia gravis and will inform future studies.

## Additional file

Additional file 1: Supplementary notes. **Table S1.** Summary of algorithms used to assess reference gene expression stability in myocytes. (I) untreated myocytes at early and late differentiation time points, (II) early untreated and MG sera (MGS) treated myocytes and (III) late untreated and MGS treated myocytes. **Figure S1.** Comparison of reference gene expression between the early and late muscle and MG models. **Table S2.** Statistical analysis of differentially expressed genes: Average Cq values and assessment of data distribution (normality testing) for differentially expressed genes. **Figure S2.** Additional genes with statistically significant (*p*<0.05) expression differences between OP-MG and control MG myocytes. (DOCX 436 kb)

## Abbreviations

∆: Delta
3’ UTR: Three prime untranslated region
AChR: Acetylcholine receptor
Cq: Threshold cycle value
CV: Coefficient of variation
DMEM: Dulbecco’s modified Eagle’s medium
EAMG: Experimental autoimmune MG
EDL: Extensor digitorum longus
EOMs: Extraocular muscles
FACS: Fluorescence-activated cell sorting
FDR: False discovery rate
GFP: Green fluorescent protein
HLA: Human leukocyte antigen
MG: Myasthenia gravis
MIQE: Minimum information for publication of quantitative real-time PCR experiments
OP-MG: Ophthalmoplegic myasthenia gravis
P/S: Penicillin streptomycin
qPCR: Quantitative PCR
RIN: RNA integrity number
ROS: Reactive oxygen species
SD: Standard deviation
WES: Whole exome sequencing

## References

[1] Rautenbach RM, Pillay K, Murray ADN, Heckmann JM. Extraocular muscle findings in myasthenia gravis associated treatment-resistant Ophthalmoplegia. J Neuro-Ophthalmology. 2017;37:414–7. 10.1097/WNO.0000000000000534 28742638

[2] Europa TA, Nel M, Heckmann JM. Myasthenic ophthalmoparesis: time to resolution after initiating immune therapies. Muscle Nerve. 2018;58:542–9. 10.1002/mus.26172 29790193

[3] Soltys J, Gong B, Kaminski HJ, Zhou Y, Kusner LL (2008). Extraocular muscle susceptibility to myasthenia gravis: unique immunological environment?. Ann N Y Acad Sci.

[4] Mombaur B, Lesosky MR, Liebenberg L, Vreede H, Heckmann JM (2015). Incidence of acetylcholine receptor-antibody-positive myasthenia gravis in South Africa. Muscle Nerve.

[5] Heckmann JM, Nel M. A unique subphenotype of myasthenia gravis. Ann N Y Acad Sci. 2018;1412:14–20. 10.1111/nyas.13471 28984362

[6] Heckmann JM, Hansen P, Van Toorn R, Lubbe E, Janse van Rensburg E, Wilmshurst JM (2012). The characteristics of juvenile myasthenia gravis among south Africans. South African Med J.

[7] Heckmann JM, Uwimpuhwe H, Ballo R, Kaur M, Bajic VB, Prince S (2010). A functional SNP in the regulatory region of the decay-accelerating factor gene associates with extraocular muscle pareses in myasthenia gravis. Genes Immun Nature Publishing Group.

[8] Nel M, Buys J, Rautenbach R, Mowla S, Prince S, Heckmann JM (2016). The African-387 C>T TGFB1 variant is functional and associates with the ophthalmoplegic complication in juvenile myasthenia gravis. J Hum Genet Nature Publishing Group.

[9] Cocuzzi ET, Bardenstein DS, Stavitsky A, Sundarraj N, Medof ME (2001). Upregulation of DAF (CD55) on orbital fibroblasts by cytokines. Differential effects of TNF-beta and TNF-alpha. Curr Eye Res.

[10] Nel M, Jalali Sefid Dashti M, Gamieldien J, Heckmann JM (2017). Exome sequencing identifies targets in the treatment-resistant ophthalmoplegic subphenotype of myasthenia gravis. Neuromuscul Disord.

[11] Fischer MD, Budak MT, Bakay M, Gorospe JR, Kjellgren D, Pedrosa-Domellöf F (2005). Definition of the unique human extraocular muscle allotype by expression profiling. Physiol Genomics.

[12] Fleige S, Pfaffl MW (2006). RNA integrity and the effect on the real-time qRT-PCR performance. Mol Asp Med.

[13] Zhou Y, Kaminski HJ, Gong B, Cheng G, Feuerman JM, Kusner L (2014). RNA expression analysis of passive transfer myasthenia supports extraocular muscle as a unique immunological environment. Investig Ophthalmol Vis Sci.

[14] Kaminski HJ, Himuro K, Alshaikh J, Gong B, Cheng G, Kusner LL (2016). Differential RNA expression profile of skeletal muscle induced by experimental autoimmune myasthenia gravis in rats. Front Physiol.

[15] Bustin SA, Benes V, Garson JA, Hellemans J, Huggett J, Kubista M (2009). The MIQE guidelines: minimum information for publication of quantitative real-time PCR experiments. Clin Chem.

[16] Stern-Straeter J, G a B, Hörmann K, Kinscherf R, Goessler UR (2009). Identification of valid reference genes during the differentiation of human myoblasts. BMC Mol Biol.

[17] Hildyard JCW, Wells DJ. Identification and Validation of Quantitative PCR Reference Genes Suitable for Normalizing Expression in Normal and Dystrophic Cell Culture Models of Myogenesis. PLoS Curr. 2014;6:1–23. 10.1371/currents.md.faafdde4bea8df4aa7d06cd5553119a6 PMC3948689 24634799

[18] Schmittgen TD, Livak KJ (2008). Analyzing real-time PCR data by the comparative CT method. Nat Protoc.

[19] Vandesompele J, De Preter K, Pattyn F, Poppe B, Van Roy N, De Paepe A (2002). Accurate normalization of real-time quantitative RT-PCR data by geometric averaging of multiple internal control genes. Genome Biol.

[20] Pfaffl MW, Tichopad A, Prgomet C, Neuvians TP (2004). Determination of stable housekeeping genes, differentially regulated target genes and sample integrity: BestKeeper--excel-based tool using pair-wise correlations. Biotechnol Lett.

[21] Masuda A, Shen X-M, Ito M, Matsuura T, Engel AG, Ohno K (2008). hnRNP H enhances skipping of a nonfunctional exon P3A in CHRNA1 and a mutation disrupting its binding causes congenital myasthenic syndrome. Hum Mol Genet.

[22] Fieller EC (1940). The biological standardization of insulin. Suppl to J R Stat Soc.

[23] MacLennan C, Beeson D, Vincent A, Newsom-Davis J (1993). Human nicotinic acetylcholine receptor alpha-subunit isoforms: origins and expression. Nucleic Acids Res.

[24] Guyon T, Levasseur P, Truffault F, Cottin C, Gaud C, Berrih-Aknin S (1994). Regulation of acetylcholine receptor alpha subunit variants in human myasthenia gravis. Quantification of steady-state levels of messenger RNA in muscle biopsy using the polymerase chain reaction. J Clin Invest.

[25] Ho JWK, Stefani M, Dos Remedios CG, Charleston MA (2008). Differential variability analysis of gene expression and its application to human diseases. Bioinformatics.

[26] Choi JK, Yu U, Yoo OJ, Kim S (2005). Differential coexpression analysis using microarray data and its application to human cancer. Bioinformatics.

[27] Bentzinger CF, Wang YX, M a R (2012). Building muscle: molecular regulation of myogenesis. Cold Spring Harb Perspect Biol.

[28] Howard JF (2018). Myasthenia gravis: the role of complement at the neuromuscular junction. Ann N Y Acad Sci.

[29] Gallegos CE, Pediconi MF, Barrantes FJ (2008). Ceramides modulate cell-surface acetylcholine receptor levels. Biochim Biophys Acta.

[30] Yanagisawa M, Nakamura K, Taga T (2004). Roles of lipid rafts in integrin-dependent adhesion and gp130 signalling pathway in mouse embryonic neural precursor cells. Genes Cells.

[31] Ohmi Y, Tajima O, Ohkawa Y, Mori A, Sugiura Y, Furukawa K (2009). Gangliosides play pivotal roles in the regulation of complement systems and in the maintenance of integrity in nerve tissues. PNAS.

[32] Ohmi Y, Tajima O, Ohkawa Y, Yamauchi Y, Sugiura Y, Furukawa K (2011). Gangliosides are essential in the protection of inflammation and neurodegeneration via maintenance of lipid rafts: elucidation by a series of ganglioside-deficient mutant mice. J Neurochem.

[33] Maurer M, Bougoin S, Feferman T, Frenkian M, Bismuth J, Mouly V (2015). IL-6 and Akt are involved in muscular pathogenesis in myasthenia gravis. Acta Neuropathol Commun.

