# Supplementary material for: Profiling of patient-specific myocytes identifies altered gene expression in the ophthalmoplegic subphenotype of myasthenia gravis
Source: Orphanet J Rare Dis. 2019 Jan 29;14:24. doi: 10.1186/s13023-019-1003-y (PMC6352355; doi:10.1186/s13023-019-1003-y)
Supplement: Supplementary file 1 — Supplementary notes. Table S1. Summary of algorithms used to assess reference gene expression stability in myocytes. (I) untreated myocytes at early and late differentiation time points, (II) early untreated and MG sera (MGS) treated myocytes and (III) late untreated and MGS treated myocytes. Figure S1. Comparison of reference gene expression between the early and late muscle and MG models. Table S2. Statistical analysis of differentially expressed genes: Average Cq values and assessment of data distribution (normality testing) for differentially expressed genes. Figure S2. Additional genes with statistically significant (p<0.05) expression differences between OP-MG and control MG myocytes. (DOCX 436 kb) [file 13023_2019_1003_MOESM1_ESM.docx]

**Additional file 1: Supplementary notes**

**Assessment of reference gene stability in myocytes**

The expression of 10 reference genes was determined in the custom qPCR array (*TFRC, HPRT1, B2M, ACTB, GUSB, RPLP0, TBP, PPIA, CSNK2A2, AP3D1*). Supplementary table 1 present the results of three methods used to comprehensively assess the stability of each reference gene: 2^-Cq^ method [1], geNorm [2] and BestKeeper [3].

Firstly, the fold change in reference gene expression induced by differentiation (where ∆Cq = reference gene Cq value in late model sample - reference gene Cq value in early model sample) and MGS (where ∆Cq = reference gene Cq value in MGS treated sample - reference gene Cq value in untreated sample) was calculated by 2^-∆Cq^ (column A). For each comparison, the Cq value of each reference gene was also expressed as 2^-∆Cq^ and these exponentiated Cq values were compared using a paired Student’s t-test to determine whether the expression of the reference gene is significantly upregulated or downregulated in response to differentiation or MGS treatment (column B) [1].

Secondly, we used the geNorm algorithm [2] implemented in the NormqPCR R package to calculate an expression stability value (m) for each gene, which represents the average pairwise variation of each individual reference gene with all other reference genes (column C). Reference genes with the lowest m value are the most stable and the suggested threshold for stability is m<0.5.

Thirdly, we used the Bestkeeper algorithm which uses a variety of metrics to evaluate expression stability [3]. Column D presents the standard deviation (SD) and coefficient of variation (CV) values which are low for genes with high expression stability. The suggested threshold for stability is SD<1.

The reference genes selected for each comparison are indicated in bold (supplementary table 1) and boxes (supplementary figure 1). *GUSB* and *TFRC* were selected to compare target gene expression levels between early and late untreated myocytes. *RPLP0* and *B2M* were selected to compare target gene expression levels between early untreated and MGS treated myocytes while *AP3D1* and *CSNK2A2* were selected to compare target gene expression levels between late untreated and MGS treated myocytes.

**Table S1.** Summary of algorithms used to assess reference gene expression stability in myocytes. (I) untreated myocytes at early and late differentiation time points, (II) early untreated and MG sera (MGS) treated myocytes and (III) late untreated and MGS treated myocytes.

| **(I) Assessing the impact of differentiation on reference gene stability** | | | | |
| --- | --- | --- | --- | --- |
| Reference Gene | A. Fold = 2^-∆Cq^  (∆Cq=5 days Cq^ref^- 48 hrs Cq^ref^)  avg fold^all^ (avg fold^control MG^ ; avg fold^OP-MG^) | B. p value (paired t test)^[1]^  5 days 2^-Cq (reference gene)^ vs  48 hrs 2^-Cq (reference gene)^ | C. geNorm^[2]^  m value | D. Bestkeeper^[3]^  SD ; CV |
| ***TFRC*** | **1.21 (1.39 ; 1.11)** | **0,177** | **0.503** | **0.29 ; 1.33** |
| *HPRT1* | 0.54 (0.59 ; 0.51) | <1x10^-4^ | 0.498 | 0.49 ; 2.01 |
| *B2M* | 1.93 (1.94 ; 1.92) | <1x10^-3^ | 0.759 | 0.48 ; 2.69 |
| *PPIA* | 0.60 (0.66 ; 0.56) | 0.001 | 0.469 | 0.44 ; 1.91 |
| *ACTB* | 0.53 (0.56 ; 0.51) | <1x10^-4^ | 0.563 | 0.51 ; 3.00 |
| ***GUSB*** | **0.96 (1.01 ; 0.92)** | **0.286** | **0.434** | **0.29 ; 1.15** |
| *RPLP0* | NA | NA | NA | NA |
| *TBP* | 0.74 (0.88 ; 0.66) | 0.020 | 0.432 | 0.43 ; 1.57 |
| *CSNK2A2* | 0.90 (1.01 ; 0.83) | 0.081 | 0.416 | 0.30 ; 1.21 |
| *AP3D1* | 0.88 (0.99 ; 0.81) | 0.085 | 0.386 | 0.28 ; 1.26 |

| **(II) Assessing the impact of MGS treatment on reference gene stability in the early model (48 hours)** | | | | |
| --- | --- | --- | --- | --- |
| Reference Gene | A. Fold = 2^-∆Cq^  (∆Cq=MGS Cq^ref^- untreated Cq^ref^)  avg fold^all^ (avg fold^control MG^ ; avg fold^OP-MG^) | B. p value (paired t test)^[1]^  MGS 2^-Cq (reference gene)^ vs untreated 2^-Cq (reference gene)^ | C. geNorm^[2]^  m value | D. Bestkeeper^[3]^  SD ; CV |
| *TFRC* | 0.91 (0.99 ; 0.86) | 0.053 | 0.366 | 0.30 ; 1.35 |
| *HPRT1* | 1.29 (1.41 ; 1.22) | 0.001 | 0.338 | 0.28 ; 1.18 |
| ***B2M*** | **1.10 (1.15 ; 1.07)** | **0.228** | **0.299** | **0.30 ; 1.62** |
| *PPIA* | 1.13 (1.23 ; 1.07) | 0.201 | 0.347 | 0.33 ; 1.47 |
| *ACTB* | 1.33 (1.40 ; 1.29) | <1x10^-3^ | 0.406 | 0.35 ; 2.16 |
| *GUSB* | 1.24 (1.35 ; 1.18) | 0.022 | 0.355 | 0.35 ; 1.36 |
| ***RPLP0*** | **1.08 (1.14 ; 1.05)** | **0.703** | **0.281** | **0.25 ; 1.51** |
| *TBP* | 1.34 (1.64 ; 1.17) | 0.084 | 0.462 | 0.45 ; 1.67 |
| *CSNK2A2* | 1.21 (1.26 ; 1.18) | 0.038 | 0.337 | 0.38 ; 1.50 |
| *AP3D1* | 1.14 (1.17 ; 1.12) | 0.035 | 0.332 | 0.36 ; 1.65 |

| **(III) Assessing the impact of MGS treatment on reference gene stability in the late model (5 days)** | | | | |
| --- | --- | --- | --- | --- |
| Reference Gene | A. Fold = 2^-∆Cq^  (∆Cq=MGS Cq^ref^- untreated Cq^ref^)  avg fold^all^ (avg fold^control MG^ ; avg fold^OP-MG^) | B. p value (paired t test)^[1]^ MGS 2^-Cq (reference gene)^ vs untreated 2^-Cq (reference gene)^ | C. geNorm^[2]^  m value | D. Bestkeeper^[3]^  SD ; CV |
| *TFRC* | 1.22 (1.21 ; 1.23) | 0.002 | 0.332 | 0.24 ; 1.09 |
| *HPRT1* | 1.09 (1.09 ; 1.09) | 0.209 | 0.269 | 0.22 ; 0.89 |
| *B2M* | 0.96 (0.98 ; 0.95) | 0.290 | 0.417 | 0.28 ; 1.62 |
| *PPIA* | 0.99 (1.01 ; 0.98) | 0.561 | 0.267 | 0.18 ; 0.75 |
| *ACTB* | 1.09 (1.05 ; 1.11) | 0.296 | 0.346 | 0.23 ; 1.31 |
| *GUSB* | 0.97 (0.99 ; 0.95) | 0.247 | 0.320 | 0.23 ; 0.88 |
| *RPLP0* | NA | NA | NA | NA |
| *TBP* | 1.12 (1.12 ; 1.12) | 0.070 | 0.285 | 0.20 ; 0.72 |
| ***CSNK2A2*** | **1.06 (1.14 ; 1.01)** | **0.852** | **0.272** | **0.19 ; 0.74** |
| ***AP3D1*** | **1.03 (1.06 ; 1.01)** | **0.945** | **0.249** | **0.17 ; 0.77** |

Fold=fold change in gene expression induced by differentiation or MGS, Cq=threshold cycle value (cycle number at which the fluorescence generated within a qPCR reaction crosses the fluorescence threshold), avg=average, MGS=MG sera, ∆=delta (difference between two Cq values), m value=expression stability value (lower value correlates with higher expression stability), SD=standard deviation, CV=coefficient of variation, NA for *RPLP0* indicates that one sample had a missing value which necessitated the exclusion of this gene as a reference candidate for a specific group. [1], [2] and [3] indicate references.

**Figure S1.** Comparison of reference gene expression between the early and late muscle and MG models.

**
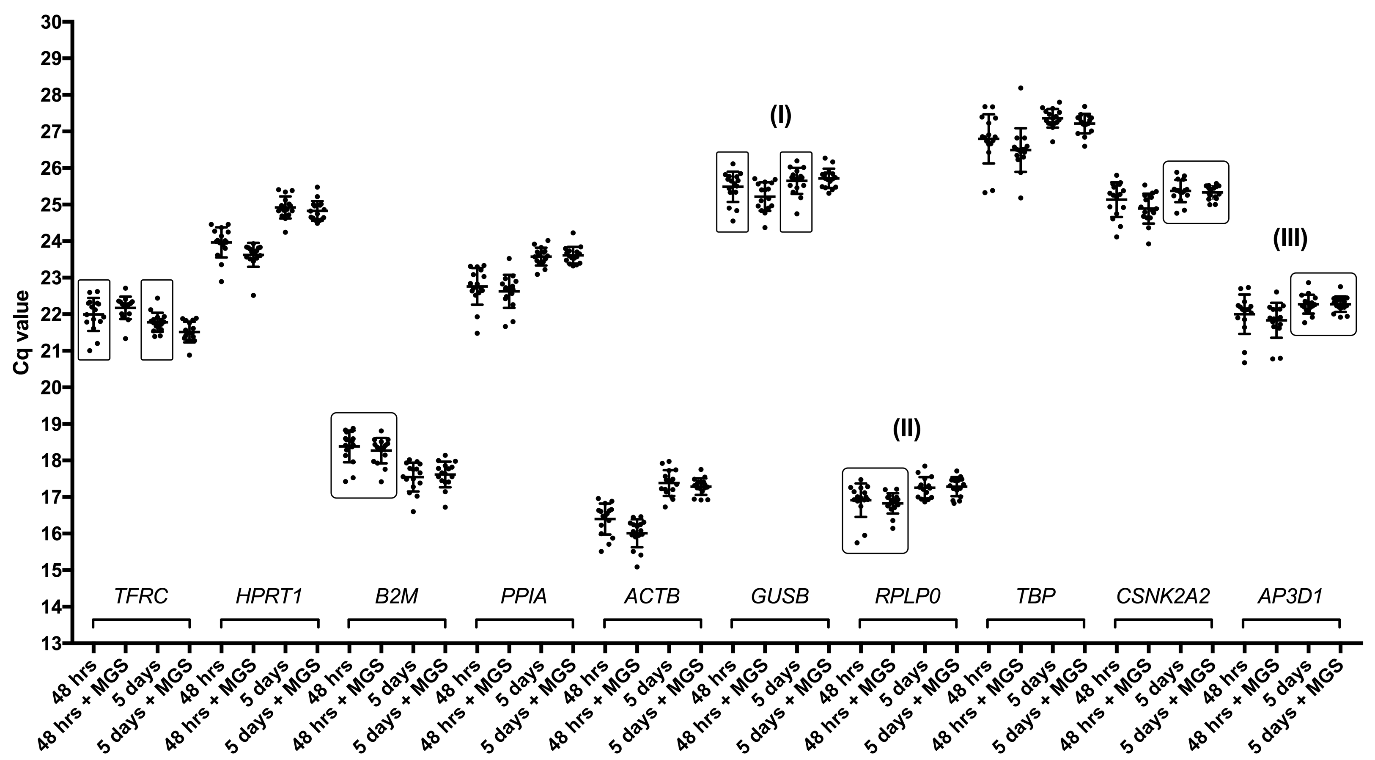
**

Individual data points indicate reference gene Cq values for 16 samples per condition (48 hrs, 48 hrs + MGS, 5 days, 5 days +MG sera). Error bars show mean and SD. Boxes indicate the reference genes selected for normalisation for each condition.

**Table S2.** Statistical analysis of differentially expressed genes**:** Average Cq values and assessment of data distribution (normality testing) for differentially expressed genes. *p<0.05; MGS refers to MG sera.

|  | **control MG (n=6)** | | | | | |  |
| --- | --- | --- | --- | --- | --- | --- | --- |
|  | **48 hrs** | | | **5 days** | | |  |
|  | average (Cq) | SD (Cq) | Shapiro-Wilk normality test (2^-∆Cq^) | average (Cq) | SD (Cq) | Shapiro-Wilk normality test (2^-∆Cq^) | condition |
| *CHRNA1* | 21.9 | 0.7 | 0.656 | 21.3 | 0.6 | 0.114 | 48 hrs vs 5 days |
| *MYOD1* | 21.8 | 0.7 | 0.491 | 22.8 | 0.5 | 0.241 | 48 hrs vs 5 days |
| *MYOG* | 33.7 | 2.0 | 0.624 | 26.5 | 1.4 | 0.010* | 48 hrs vs 5 days |
|  | **OP-MG (n=10)** | | | | | |  |
|  | **48 hrs** | | | **5 days** | | |  |
|  | average (Cq) | SD (Cq) | Shapiro-Wilk normality test (2^-∆Cq^) | average (Cq) | SD (Cq) | Shapiro-Wilk normality test (2^-∆Cq^) | condition |
| *CHRNA1* | 21.4 | 0.7 | 0.059 | 21.0 | 0.4 | 0.614 | 48 hrs vs 5 days |
| *MYOD1* | 21.5 | 0.6 | 0.416 | 22.6 | 0.3 | 0.421 | 48 hrs vs 5 days |
| *MYOG* | 32.7 | 0.7 | 0.165 | 27.2 | 2.8 | 0.263 | 48 hrs vs 5 days |

|  | **control MG (n=6)** | | | | | |  |
| --- | --- | --- | --- | --- | --- | --- | --- |
|  | **untreated** | | | **MGS** | | |  |
|  | average (Cq) | SD (Cq) | Shapiro-Wilk normality test (2^-∆Cq^) | average (Cq) | SD (Cq) | Shapiro-Wilk normality test (2^-∆Cq^) | condition |
| *ANGPTL4* | 28.4 | 0.8 | 0.455 | 25.9 | 0.4 | 0.863 | 48 hrs MGS vs untreated |
| *SPHK1* | 25.0 | 0.3 | 0.315 | 23.7 | 0.3 | 0.464 | 48 hrs MGS vs untreated |
| *SMAD3* | 28.9 | 0.4 | 0.290 | 27.9 | 0.6 | 0.205 | 48 hrs MGS vs untreated |
|  | **OP-MG (n=10)** | | | | | |  |
|  | **untreated** | | | **MGS** | | |  |
|  | average (Cq) | SD (Cq) | Shapiro-Wilk normality test (2^-∆Cq^) | average (Cq) | SD (Cq) | Shapiro-Wilk normality test (2^-∆Cq^) | condition |
| *ANGPTL4* | 28.2 | 1.3 | 0.135 | 26.2 | 0.9 | 0.157 | 48 hrs MGS vs untreated |
| *SPHK1* | 24.8 | 0.9 | 0.816 | 24.0 | 1.3 | 0.574 | 48 hrs MGS vs untreated |
| *SMAD3* | 29.7 | 1,4 | 0.750 | 28.5 | 1.0 | 0.291 | 48 hrs MGS vs untreated |

|  | **control MG (n=6)** | | | **OP-MG (n=10)** | | |  |
| --- | --- | --- | --- | --- | --- | --- | --- |
|  | average (Cq) | SD (Cq) | Shapiro-Wilk normality test (2^-∆Cq^) | average (Cq) | SD (Cq) | Shapiro-Wilk normality test (2^-∆Cq^) | condition |
| *UCP3* | 32.1 | 0.7 | 0.109 | 32.7 | 0.9 | 0.983 | 48 hrs untreated |
| *PPP6R2* | 26.0 | 0.2 | 0.504 | 26.2 | 0.6 | 0.008* | 48 hrs untreated |
| *CANX* | 19.8 | 0.1 | 0.888 | 20.0 | 0.4 | 0.002* | 48 hrs MGS |
| *PAX3* | 25.5 | 0.6 | 0.236 | 25.9 | 0.5 | 0.905 | 48 hrs MGS |
| *SPTLC1* | 23.3 | 0.2 | 0.303 | 23.4 | 0.5 | 0.132 | 48 hrs MGS |
| *UGCG* | 21.6 | 0.3 | 0.200 | 21.7 | 0.2 | 0.580 | 48 hrs MGS |
| *ACSL5* | 27.9 | 0.7 | 0.903 | 28.8 | 0.8 | 0.274 | 48 hrs MGS |
| *CD55* | 22.7 | 0.5 | 0.120 | 22.1 | 0.5 | 0.603 | 48 hrs MGS |
| *BAFF* | 30.6 | 0.8 | 0.947 | 31.2 | 0.8 | 0.916 | 48 hrs MGS |
| *IL6ST* | 21.0 | 0.3 | 0.827 | 21.1 | 0.3 | 0.596 | 48 hrs MGS |
| *AKT2* | 22.4 | 0.2 | 0.663 | 22.5 | 0.4 | 0.001* | 48 hrs MGS |
| *MAP4K5* | 23.2 | 0.3 | 0.082 | 23.2 | 0.3 | 0.971 | 48 hrs MGS |
| *FAM136A* | 24.7 | 0.2 | 0.759 | 25.0 | 0.4 | 0.005* | 5 days untreated |
| *FAM69A* | 22.2 | 0.2 | 0.463 | 22.0 | 0.3 | 0.645 | 5 days MGS |

**Figure S2. Control MG and OP-MG myocytes show different gene expression profiles.**

**
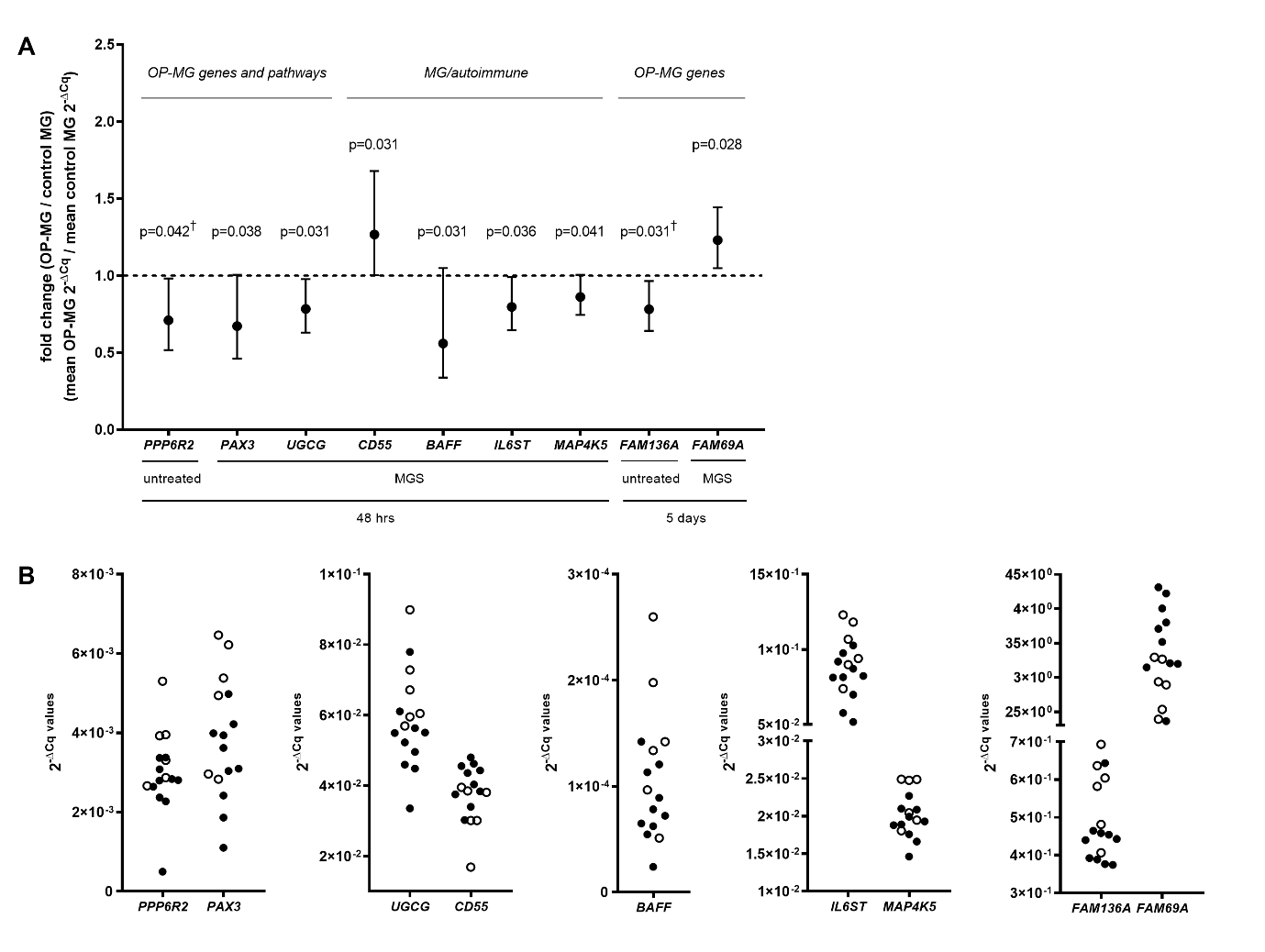
**

RNA was extracted from untreated and MG sera (MGS) treated control MG (n=6) and OP-MG (n=10) myocytes after 48 hours and 5 days differentiation as described. Target gene expression levels were determined using the custom qPCR gene expression array and a fold change in gene expression (OP-MG/control MG) was calculated for each gene in each of the four models: early and late muscle models (untreated) and early and late MG models (MGS treated). Genes with statistically significant (p<0.05) fold changes which are not shown in Fig. 1 are shown here. A. shows the fold change as an average of OP-MG/control MG samples (error bars show mean and 95% CI) and B. shows the 2^-∆Cq^ values for each sample (open circles=control MG, closed circles=OP-MG). Student’s t test was used for comparisons where the data was normally distributed, otherwise Mann-Whitney test was used (†) where Shapiro-Wilk normality test p<0.05.

**References**

1. Schmittgen TD, Livak KJ. Analyzing real-time PCR data by the comparative CT method. Nat Protoc. 2008;3:1101–8.

2. Vandesompele J, De Preter K, Pattyn F, Poppe B, Van Roy N, De Paepe A, et al. Accurate normalization of real-time quantitative RT-PCR data by geometric averaging of multiple internal control genes. Genome Biol. 2002;3:RESEARCH0034.

3. Pfaffl MW, Tichopad A, Prgomet C, Neuvians TP. Determination of stable housekeeping genes, differentially regulated target genes and sample integrity: BestKeeper--Excel-based tool using pair-wise correlations. Biotechnol Lett. 2004;26:509–15.
